# Acute adrenal necrosis in a young female cat

**DOI:** 10.1111/jvim.16926

**Published:** 2023-11-09

**Authors:** Rebecca A. Manson, Tara N. Hammond, Julie E. Callahan Clark

**Affiliations:** ^1^ Small Animal Internal Medicine Service University of Georgia Veterinary Teaching Hospital Athens Georgia USA; ^2^ Emergency and Critical Care Service Tufts Veterinary Emergency Treatment & Specialties Walpole Massachusetts USA; ^3^ Internal Medicine Service Tufts Veterinary Emergency Treatment and Specialties Walpole Massachusetts USA

**Keywords:** acute adrenal necrosis, Addison's disease, adrenomegaly, feline, hypoadrenocorticism

## Abstract

**Case Description:**

An 18‐month‐old spayed female domestic short haired cat was presented for poor appetite, lethargy, exaggerated swallowing, and regurgitation 2 weeks after endoscopic retrieval of gastric foreign material.

**Clinical Findings:**

The cat was quiet with tacky mucous membranes on physical examination. Point‐of‐care blood testing identified mild azotemia, moderate hypercalcemia, and a sodium‐to‐potassium ratio of 26. An ultrasound examination the next day identified moderate to marked bilateral adrenomegaly. Cytology of a fine needle aspirate of the adrenal glands was consistent with necrosis and associated inflammation. Hypoadrenocorticism was diagnosed by a confirmatory adrenocorticotropic hormone stimulation test.

**Treatment and Outcome:**

The cat normalized both clinically and biochemically after treatment with prednisolone and desoxycorticosterone pivalate.

**Clinical Relevance:**

Acute adrenal necrosis has been well documented in human medicine after anesthetic events. To our knowledge, hypoadrenocorticism caused by cytologically confirmed acute adrenal necrosis has not been previously reported in dogs and cats.

**Abbreviations**
ACTH: adrenocorticotropic hormone
DOCP: desoxycorticosterone pivalate

## 1 INTRODUCTION

Hypoadrenocorticism is a deficiency of the hormones produced by the adrenal glands. Without these hormones, in particular corticosteroids and mineralocorticoids, the body cannot appropriately respond to increased physiologic stress or regulate serum electrolyte concentrations. Hypoadrenocorticism in cats is uncommon, with approximately 40 cases described in the literature.^1^, ^2^, ^3^, ^4^, ^5^, ^6^, ^7^, ^8^, ^9^, ^10^, ^11^, ^12^, ^13^, ^14^, ^15^, ^16^, ^17^

The etiology of hypoadrenocorticism in dogs is believed to be immune‐mediated lymphoplasmacytic inflammation and adrenocortical atrophy. Antibodies to adrenal cell antigens have been detected in 2 dogs with primary hypoadrenocorticism.^18^, ^19^ The etiology of hypoadrenocorticism in cats remains largely unknown. Two cases of trauma‐induced hypoadrenocorticism and 3 cases of primary adrenal lymphoma causing hypoadrenocorticism have been reported in cats.^2^, ^5^, ^6^, ^16^

Limited data is available on the ultrasonographic appearance of the adrenal glands in cats with primary hypoadrenocorticism. In the few cases in which the ultrasonographic appearance has been documented, the adrenal glands have been normal in size except for a case report in which the right adrenal gland could not be identified.^8^, ^14^, ^15^, ^17^ Adrenal insufficiency with adrenomegaly previously has been documented in cats only in the context of primary adrenal lymphoma.^2^, ^16^

In a study of 33 dogs with adrenalitis, inflammation was found to be lymphoplasmacytic in 17, lymphohistiocytic in 1, lymphocytic in 4, granulomatous in 3, and neutrophilic in 8. Adrenocortical atrophy was associated primarily with lymphoplasmacytic inflammation as opposed to non‐lymphoid inflammation.^19^ Inflammatory adrenal disease has not been reported previously in cats.

A single case report of a dog with bilateral adrenomegaly and adrenocortical necrosis with neutrophilic inflammation on adrenal cytology has been described. The affected dog had an adrenocorticotropic hormone (ACTH) stimulation performed that confirmed hypoadrenocorticism 12 days after being diagnosed with hyperadrenocorticism by an ACTH stimulation test. Treatment was not initiated. The dog died suddenly 10 months after diagnosis and a pituitary chromophore adenoma was identified on necropsy. The adrenal glands were small and fibrotic at necropsy.^20^

## 2 CASE DESCRIPTION

An 18‐month‐old spayed female domestic short haired cat, weighing 3.6 kg, was presented for evaluation of progressive vomiting and decreased appetite for 48 hours. The cat was previously healthy and current on routine medical care. Physical examination was unremarkable aside from a distended abdomen. Linear to tubular coalescing soft‐tissue opacities were evident in the stomach on 2‐view abdominal radiographs. No discrete foreign material was visualized in the small intestine or colon. The remaining organs were unremarkable. A CBC and serum biochemistry profile were unremarkable. The sodium‐to‐potassium ratio was 41.

The cat was hospitalized overnight and treated with IV lactated Ringer's solution (80 mL/kg/day) and maropitant (1 mg/kg IV q24). The next day the cat was anesthetized for endoscopic removal of the gastric foreign material. It was pre‐medicated with methadone (0.2 mg/kg IV) and ketamine (1 mg/kg IV). Anesthesia was induced with propofol (3.6 mg/kg IV), and the cat was intubated and maintained with isoflurane. At surgery, several fabric hair ties were removed from the stomach. Systolic blood pressure remained normal (80‐115 mm Hg) throughout the procedure. The cat was discharged later that night with no medications.

Two weeks later, the cat was re‐presented because of progressively poor appetite, dysphagia, and regurgitation. The owner reported that the cat did well for the first few days after endoscopy, but appetite had steadily declined and the cat was now anorexic. On presentation the cat was quiet, tachycardic (220 beats per minute [bpm]), and intermittently exhibited exaggerated swallowing. The cat was estimated to be 5% dehydrated with pale pink, tacky mucous membranes. Abdominal palpation was normal. Abdominal radiography was unremarkable. Point‐of‐care blood testing identified hyponatremia (137.1 mmol/L [normal, 146.2‐156.2 mmol/L]), hyperkalemia (5.32 mmol/L [normal, 3.41‐4.71 mmol/L]), ionized hypercalcemia (1.76 mmol/L [normal, 1.16‐1.35 mmol/L]), and mild azotemia (serum creatinine concentration 2.22 mg/dL [normal, 0.70‐1.90 mg/dL] and BUN 51 mg/dL [normal, 22‐33 mg/dL]). The sodium‐to‐potassium ratio was 26. The cat was hemoconcentrated with a packed cell volume of 55% and total protein concentration of 9.0 g/dL. Urinalysis obtained by cystocentesis indicated a urine specific gravity of 1.038 and 1+ proteinuria. The cat was admitted to the hospital for treatment of suspected esophageal disease based on the presence of regurgitation and dysphagia after recent endoscopy. A 20 mL/kg IV fluid bolus was administered, and fluid therapy then was continued with lactated Ringer's solution at 90 mL/kg/day. Treatment was initiated with pantoprazole (1 mg/kg IV q12) and maropitant (1 mg/kg IV q24).

The next day the cat remained quiet, but the tachycardia had resolved (160 bpm). Intermittent muscle fasciculations and bilateral mydriasis were observed. Systolic blood pressure was normal at 120 mm Hg. Recheck point‐of‐care laboratory testing identified ionized hypercalcemia of 1.97 mmol/L (normal, 1.16‐1.35 mmol/L). The serum sodium concentration remained low (141 mmol/L [normal, 146.2‐156.2 mmol/L]) and serum potassium concentration remained in the high normal range (4.71 mmol/L [normal, 3.41‐4.71 mmol/L]). Azotemia had resolved. A CBC was normal but lacked a stress leukogram. A serum biochemistry profile identified total hypercalcemia (14.6 mg/dL [normal, 8.2‐11.2 mg/dL]), hypocholesterolemia (49 mg/dL [normal, 91‐305 mg/dL]), and low normal serum albumin concentration (2.8 g/dL [normal, 2.6‐3.9 g/dL]). Because of progressive hypercalcemia, fluid therapy was changed from lactated Ringer's solution to 0.9% NaCl.

**FIGURE 1:**
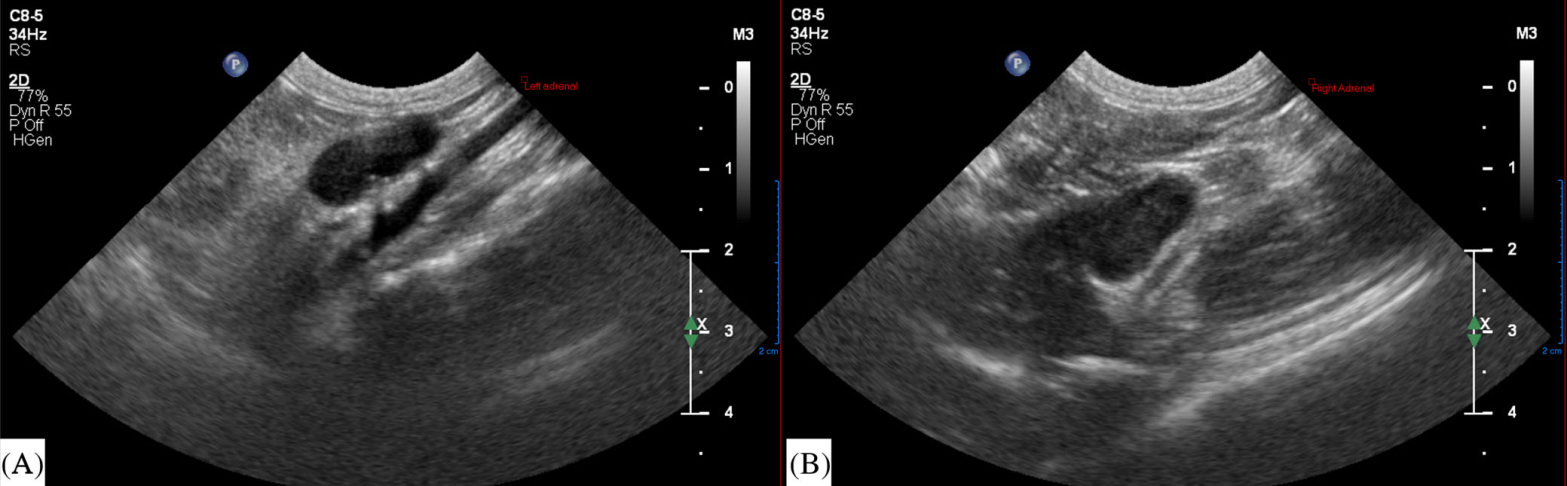
Ultrasonographic images of bilaterally enlarged, hypoechoic adrenal glands. (A) Left adrenal gland, 2 cm scale bar. (B) Right adrenal gland, 2 cm scale bar.

An abdominal ultrasound examination determined that the adrenal glands were hypoechoic and moderately to markedly enlarged bilaterally, each measuring up to 1 cm in thickness (Figure 1). Ultrasound‐guided fine needle aspirates of the adrenal glands were performed. After the procedure, an ACTH stimulation test was performed. After collection of the post‐ACTH blood sample the cat was given dexamethasone (0.2 mg/kg IV q24). Pantoprazole and maropitant were continued.

**FIGURE 2:**
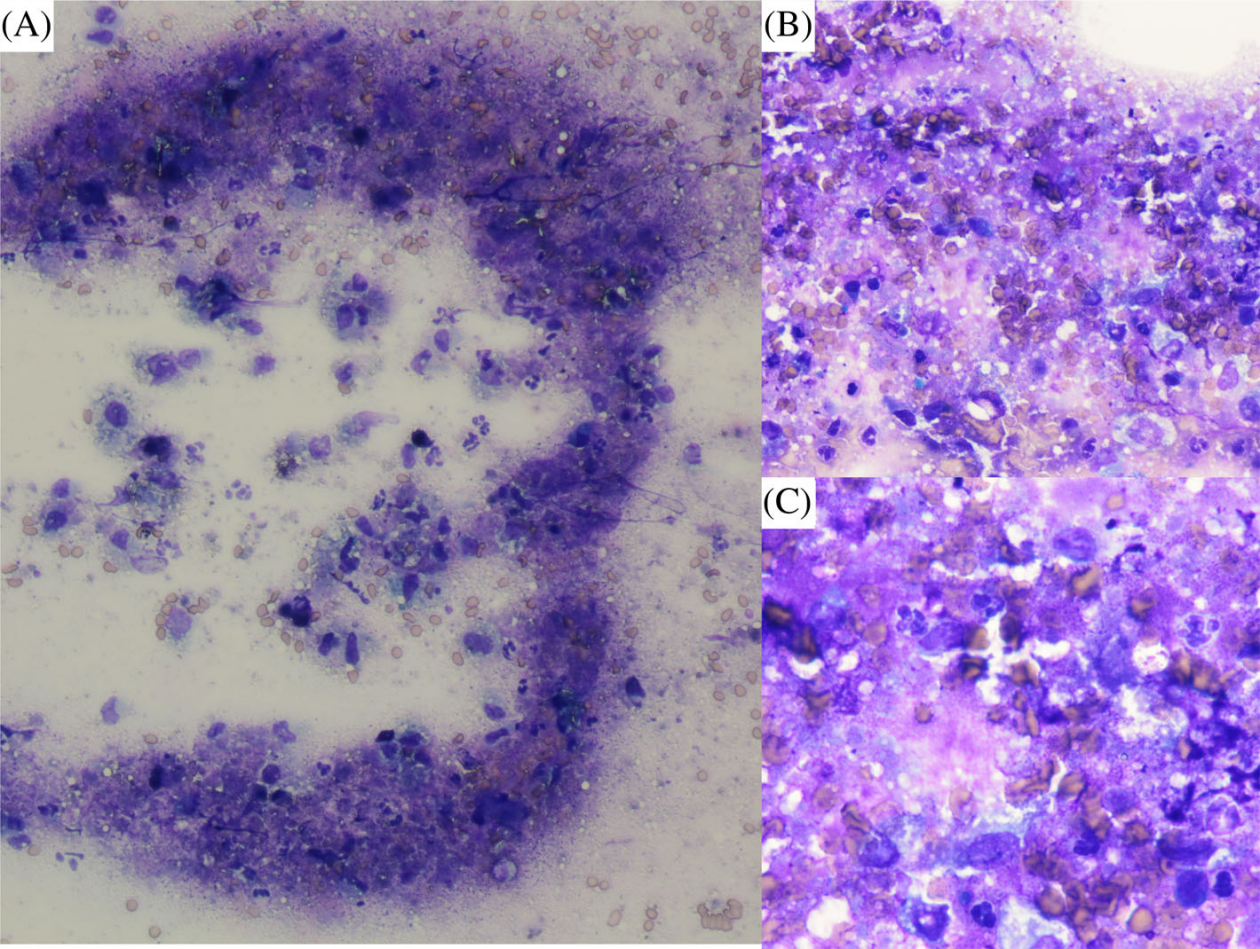
Photomicrograph of adrenal gland fine needle aspirate demonstrating necrosis with neutrophilic/macrophagic inflammation. (A) 20×, 50 μm scale bar. (B) 50×, 20 μm scale bar. (C) 100×, 20 μm scale bar. Slides were stained with a Wescor Aerospray Hematology Stat slide stainer using Fisherbrand Hemaspray reagents.

By the next morning the cat was bright and eating. The muscle fasciculations and bilateral mydriasis had resolved. Point‐of‐care laboratory testing identified markedly improved ionized serum calcium concentration of 1.39 mmol/L (normal, 1.16‐1.35 mmol/L). The sodium‐to‐potassium ratio also was improved (36) with serum sodium concentration of 154.3 mmol/L (normal, 146.2‐156.2 mmol/L) and serum potassium concentration of 4.20 mmol/L (normal, 3.41‐4.71 mmol/L). The ACTH stimulation test results confirmed hypoadrenocorticism (pre‐ACTH cortisol concentration < 0.2 mg/dL, post‐ACTH cortisol concentration < 0.2 mg/dL). Cytology of the adrenal glands was consistent with necrosis with associated neutrophilic and macrophagic inflammation (Figure 2). Blood contamination and hemorrhage were also evident. No evidence of neoplasia was identified.

Before discharge from the hospital the cat was started on prednisolone (0.5 mg/kg PO q24) and desoxycorticosterone pivalate (DOCP, 2.2 mg/kg IM). After 5 days at home, the prednisolone dosage was decreased to 0.25 mg/kg PO q24 with instructions to double the dose during times of stress.

The cat returned for follow‐up 14 days after receiving the first dose of DOCP. At that time the owner reported that the cat was almost completely back to normal. Serum electrolyte concentrations were stable (ionized calcium concentration 1.37 mmol/L [normal, 1.16‐1.35 mmol/L], serum potassium concentration 4.97 mmol/L [normal, 3.41‐4.71 mmol/L], serum sodium concentration 151.3 mmol/L [normal, 146.2‐156.2 mmol/L], Na:K ratio 30). Over the next 6 months, the interval of DOCP administration was gradually shortened from 30 to 22 days because of persistently low sodium‐to‐potassium ratio on the day of injection coupled with owner‐reported lethargy for several days leading up to the scheduled injection. The owner was instructed to administer DOCP at home every 21‐24 days depending on the cat's clinical signs. Prednisolone was continued at a dosage of 0.25 mg/kg PO q24. The cat was doing well 14 months after diagnosis with this treatment protocol.

## 3 DISCUSSION

To the best of our knowledge, this case report represents the first example of inflammatory adrenomegaly associated with adrenal insufficiency in a cat. Hemorrhage, necrosis, and neutrophilic inflammation were noted on cytology, and it is unclear which process represents the primary insult. The neutrophilic nature of the inflammation suggests that it was not an immune‐mediated process.

Adrenal insufficiency because of acute adrenal hemorrhage and subsequent necrosis previously has been reported in humans in times of increased physiological stress (characterized by increased ACTH release and hypercortisolism) such as recent surgery, hypotension, and sepsis. This phenomenon has not been evaluated in veterinary patients. Aside from the anesthetic event, and associated brief hospitalization, no other risk factors were identified in this case.^20^, ^21^, ^22^, ^23^ The cat was previously healthy and underwent anesthesia 2 weeks before being diagnosed with hypoadrenocorticism. There was no evidence of sepsis either clinically or on pre‐anesthetic laboratory testing. No anti‐coagulants were used. Blood pressure remained normal throughout the short anesthetic event.

The other reported case of acute neutrophilic adrenalitis and necrosis occurred in a dog with a functional pituitary tumor after an ACTH stimulation test was performed to diagnose hyperadrenocorticism.^24^ A study performed on rats concluded that administration of high doses of ACTH (60 μg) lead to adrenal hemorrhage, apoptosis and vacuolization.^25^ The proximity in time between the anesthetic event and diagnosis of hypoadrenocorticism in the case reported here raises the question of whether increased stress at the time of anesthesia could have caused adrenal necrosis. This scenario would suggest that the cat's endogenous ACTH concentration was at some point very high which, in the absence of a measured endogenous ACTH concentration at the time of anesthesia, cannot be evaluated. Although the use of etomidate for induction of anesthesia has been associated with a transient decrease in cortisol synthesis in cats, etomidate was not utilized during this anesthetic protocol.^26^ Ketamine, which was used during this cat's anesthesia, has been shown to transiently inhibit the release of ACTH in fetal sheep, but this effect has not been documented in cats.^27^, ^28^

Ultimately, the underlying cause of this cat's adrenal disease is unclear. It is possible that ingestion of an unknown toxin or release of a toxin from the breakdown of hair tie elastics could have led to the adrenal hemorrhage and necrosis. Emerging research on endocrine disrupting chemicals suggests a possible role in adrenal disease.^29^

The cat described here represents the second report of adrenal insufficiency in the face of the bilateral adenomegaly and necrosis, and the first such case in a cat. We describe a previously unreported cause of hypoadrenocorticism in cats and illustrate that large adrenal glands on ultrasound examination do not rule out hypoadrenocorticism as a diagnosis.

## CONFLICT OF INTEREST DECLARATION

Authors declare no conflict of interest.

## OFF‐LABEL ANTIMICROBIAL DECLARATION

Authors declare no off‐label use of antimicrobials.

## INSTITUTIONAL ANIMAL CARE AND USE COMMITTEE (IACUC) OR OTHER APPROVAL DECLARATION

Authors declare no IACUC or other approval was needed.

## HUMAN ETHICS APPROVAL DECLARATION

Authors declare human ethics approval was not needed for this study.
